# Characteristics of pigmentary glaucoma in Japanese individuals

**DOI:** 10.1371/journal.pone.0268864

**Published:** 2022-06-23

**Authors:** Takehiro Yamashita, Hideki Shiihara, Hiroto Terasaki, Kazuki Fujiwara, Minoru Tanaka, Taiji Sakamoto

**Affiliations:** Department of Ophthalmology, Kagoshima University Graduate School of Medical and Dental Sciences, Kagoshima, Japan; Massachusetts Eye & Ear Infirmary, Harvard Medical School, UNITED STATES

## Abstract

**Purpose:**

Myopia is a known risk factor of pigmentary glaucoma (PG), and the increased prevalence of myopia in Asian countries indicates that more cases of PG will likely develop soon. However, there are no diagnostic criteria for PG for Asians. Therefore, the purpose of this study is to determine the characteristics of PG in Japanese individuals and establish three diagnostic signs for PG.

**Methods:**

This was a single-center, retrospective, case series study of glaucoma patients who visited the Kagoshima University Hospital between January 2015 and January 2020. The inclusion criteria were age <50 years at time of diagnosis and presence of pigmentation in the anterior chamber (AC) angle including a Sampaolesi line. Eyes with pigmentation of the AC angle caused by other types of glaucoma such as uveitis, trauma, exfoliation, or childhood glaucoma were excluded. We investigated the classic diagnostic triad of signs of PG; posterior corneal pigmentation, mid peripheral iris transillumination defect, and pigmentation of the trabecular meshwork. We also examined the Sampaolesi line, iris concavity, and midperipheral iris depigmentation in eyes with PG.

**Results:**

Ten eyes of 5 Japanese men and 10 eyes of 5 Japanese women were studied. Their age ranged from 13 to 46 years at the time of diagnosis. One eye had posterior corneal pigmentation and 6 eyes had pigmentation of the trabecular meshwork. None had mid peripheral iris transillumination defect. The Sampaolesi line, iris concavity, and midperipheral iris depigmentation were found in all patients except one patient who lacked the mid peripheral depigmentation. Two eyes had the pigment reversal sign, none had lens pigmentation, and 2 eyes had peripheral retinal degeneration.

**Conclusion:**

The presence of the Sampaolesi line, iris concavity, and midperipheral iris depigmentation may be appropriate signs for the diagnosis of PG in Asians.

## Introduction

Pigmentary glaucoma (PG) was first described by Sugar and Barbour in 1949. The diagnostic triad of signs for PG were slit-like mid peripheral iris transillumination defect, diffuse and dense brownish pigmentation of the anterior chamber (AC) angle, and pigment granules on the corneal endothelium (Krukenberg spindle). When the triad was not accompanied by glaucoma, the clinical entity was named the pigment dispersion syndrome (PDS) [1].

The clinical course of PG can be divided into three phases. The first phase is the active pigment dispersion phase, and it can begin as early as elementary school age. The intraocular pressure (IOP) is normal in this phase. In the second phase is the conversion to glaucoma phase and is when the triad of signs develop, and the individual is diagnosed with PG. The third phase is the regression or self-healing phase when the IOP recovers to within the normal limits and the pigmentation begins clearing from the trabecular meshwork. The AC angle pigmentation will appear denser and darker in the lower than in the upper angle with active pigment dispersion. However, the pigment will appear much denser and darker superiorly than inferiorly in the self-healing phase. This pigment reversal sign can be detected whenever the pigment has been spontaneously cleared from the trabecular meshwork. Finally, the patient can be labeled as having normal tension glaucoma (NTG) [2, 3].

The typical patient with PG is young, myopic, and male. On the other hand, the individual with angle closure glaucoma is old, hyperopic, and female [2, 3]. PG and PDS have been thought to be rare in the Asian population [4], but it is interesting that the prevalence of myopia is high and is increasing in the younger generation. In addition, the prevalence of NTG is high in Asians and even higher than that in Caucasians [5, 6]. Thus, we hypothesized that the NTG patients in Japan may include some individuals with PG in the regression phase.

Originally, the diagnostic criteria for open angle glaucoma (OAG), including NTG, were that there were no abnormalities in the AC angle. However even if there was dense pigmentation in the angle, OAG would be diagnosed because of the absence of the triad of pathognomonic signs of PG such as the mid peripheral iris transillumination defect [2].

**Fig 1:**
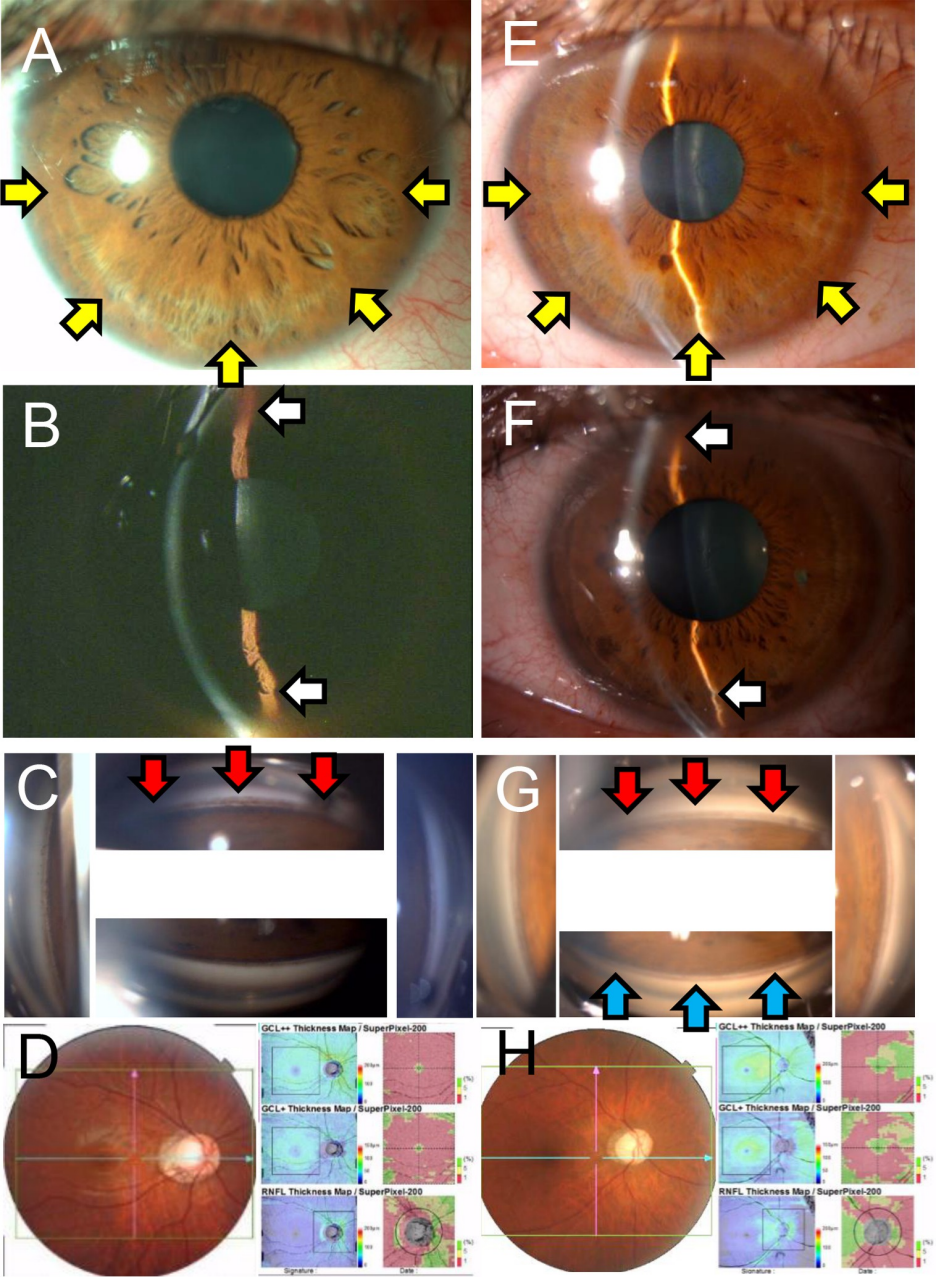
Representative cases of pigmentary glaucoma in Japanese subjects. Slit-lamp and gonioscopic images, and fundus photographs of the right eyes of a 28-year-old man with pigmentary glaucoma (PG) (Case 4, A, B, C, D) and a 45-year-old man (Case 9, E, F, G, H) with PG. Both were Japanese. Midperipheral iris depigmentation (yellow arrows) and iris concavity (white arrows) can be seen in the slit-lamp images. Dense pigmentation of the trabecular meshwork and Schwalbe line (red arrows) can be seen in the gonioscopic images. The pigment reversal sign (upper >lower pigmentation; blue arrows) can be seen in the 45-year-old case.

Slit-lamp and gonioscopic images, and fundus photographs of a 28-year-old PG patient and a 45-year-old PG patient are shown in Fig 1. Both patients were Japanese and had dense pigmentation of the AC angle but did not have mid peripheral iris transillumination defect and Krukenberg spindle. Both were diagnosed with open angle glaucoma at the previous hospital. However, mid peripheral iris depigmentation and iris concavity were seen in slit-lamp examinations.

The classic triad of signs for PG was developed for Caucasian individuals with PG at the conversion to glaucoma and active pigment dispersion phases. However, all of the triad of signs were not seen in the regression phase even in Caucasians [2]. In addition, the mid peripheral iris transillumination defect sign is difficult to detect in the thick iris of Asian eyes. Therefore, we need new signs for PG and PDS in Asians.

Thus, the purpose of this study was to determine the characteristics of eyes with PG in Japanese individuals and to determine whether there are signs in patients with PG that can be used to diagnose PG accurately.

## Methods

All of the procedures used conformed to the tenets of the Declaration of Helsinki. This study was approved by the Ethics Committee of Kagoshima University Hospital (approval number 190314). The need for informed consent was waived by the Ethics Committee because of the retrospective nature of the study. We published the research plan on the homepage of our hospital’s website and guaranteed an opt-out opportunity according to the instructions of the IRB. All data were fully anonymized before analysis.

This was a single-center, retrospective, case series study of glaucoma patients who were examined at the Kagoshima University Hospital between January 2015 and January 2020. The inclusion criteria were age <50 years at the time of diagnosis and the presence of pigmentation in the AC angle including the Sampaolesi line, a pigmentation of the Schwalbe line. Those with pigmentation of the AC angle associated with other types of glaucoma such as uveitis, trauma, exfoliation, or childhood glaucoma were excluded.

All patients underwent auto refractometer, slit-lamp biomicroscopy, non-dilated and dilated ophthalmoscopy, gonioscopy, optical coherence tomography (OCT), IOP measurements by Goldmann tonometry, and visual fields by Humphrey SITA standard 30–2 or by Goldmann perimetry. We investigated the classic diagnostic triad of signs for PG including posterior corneal pigmentation, mid peripheral iris transillumination defect, and pigmentation on the trabecular meshwork. We also determined whether other findings such as Sampaolesi line, pigment reversal sign, iris concavity, midperipheral iris depigmentation, pigmentation of lens, and peripheral retina degeneration were present [2]. The diagnosis was made by at least two examiners for each eye and an agreement was obtained for every eye.

## Results

A total of 1289 charts of patients who were examined in the glaucoma clinic of the Kagoshima University Hospital between January 2015 to January 2020 were studied. Ten eyes of 5 Japanese men and 10 eyes of 5 Japanese women met the inclusion criteria. Their ages ranged from 13 to 46 years at the time of the diagnosis of PG. The demographic information of these 10 participants is shown in Table 1. Case 2 was diagnosed with PDS because the OCT findings were normal. The other 9 eyes were diagnosed with PG with glaucomatous visual field defects. All ten patients were myopic. An IOP >21 mmHg was present only in the 3 teenage patients.

**Table 1:** Demographic information and ocular characteristics of the participants.

Case number	Sex	Age of diagnosis	Refractive error in right eye	Refractive error in left eye	IOP in right eye	IOP in left eye	MD value in right eye	MD value in left eye
1	Female	13	-3.25	-3.25	25	25	-3.5	-4.53
2	Female	15	-12	-10.25	27	27	Normal OCT	Normal OCT
3	Male	15	-8.5	-8.25	26	28	-7.9	-21.42
4	Male	28	-3.75	-3.75	15	16	-17.7	GP Vb
5	Female	31	-5.5	-4.75	19	19	-2.61	-3.6
6	Male	36	-4.5	-4.25	14	14	-5.08	-3.03
7	Female	42	-5.75	-6.25	14	13	-9.39	-3.42
8	Female	42	-7.25	-7	Unknown	Unknown	-15.7	-16.37
9	Male	45	-23	-10	20	20	-11.08	-5.85
10	Male	46	-6	-6.75	Unknown	Unknown	-9.43	-9.24

IOP, intraocular pressure in mmHg; MD, mean deviation of Humphrey perimetry; OCT, optical coherence tomography; GP, Goldmann perimetry.

One eye had posterior corneal pigmentation and 6 eyes had pigmentation on the trabecular meshwork. None had mid peripheral iris transillumination defect. The Sampaolesi line and iris concavity were found in all patients, and midperipheral iris depigmentation was found in 9 patients. Two patients had the pigment reversal sign, none had lens pigmentation, and 2 had peripheral retinal degenerations. Both eyes of each individual had similar characteristics (Table 2).

**Table 2:** Classic diagnostic triad and other findings of patients with pigmentary glaucoma.

Case number	Pigmentation of cornea	Pigmentation of trabecular meshwork	Midperipheral iris transillumination defect	Sampaolesi line	Pigment reversal sign	Iris concavity	Mid peripheral iris depigmentation	Pigmentation of lens	Peripheral retinal degeneration
1		O		O		O	O		
2				O		O			O
3		O		O		O	O		
4	O	O		O		O	O		
5		O		O		O	O		
6				O		O	O		
7		O		O	O	O	O		
8				O		O	O		
9		O		O	O	O	O		O
10				O		O	O		

## Discussion

Our results showed that the percentage of patients with some of the classic triad of signs for PG and PDS was 10% for posterior corneal pigmentation, 0% for mid peripheral iris transillumination defect, and 60% for pigmentation of the trabecular meshwork. The posterior corneal pigmentation and pigmentation of the trabecular meshwork were seen only during the active pigment dispersion phase and were appropriate diagnostic signs for only young patients. However, they were not appropriate for elderly individuals in which the pigmentation was absorbed and not present during the regression phase when the pigment dispersion had subsided.

Mid peripheral iris transillumination defect was not present in any of the cases. Because Asians have thicker irises with denser pigmentation than Caucasians [7], the mid peripheral iris transillumination defect may not be detected unless there is a high degree of pigment dispersion [4]. In addition, because there are individual differences in the iris thickness and pigmentation even among Asians [8], the mid peripheral iris transillumination defect will probably be present only if pigment dispersion occurs in eyes with thin and lower density of pigmentation in the iris. A retrospective study of 21 Chinese patients with PDS or PG reported that all of the patients had pigmentation on the trabecular meshwork and posterior corneal pigmentation (Krukenberg spindle) [4]. We assumed that these two signs were used for the diagnosis of PDS and PG because mid peripheral iris transillumination defect was found in only one patient. These findings are consistent with our results. We believe that mid peripheral iris transillumination defect rarely occurs in the eyes of Asians, and it is not a suitable diagnostic sign for PG in Asians.

On the other hand, the findings in our cohort were the Sampaolesi line in 100%, iris concavity in 100%, and mid peripheral iris depigmentation in 90%. In the regression phase, the pigments in the trabecular meshwork were absorbed from the lower part of the eye resulting in the pigment reversal sign (Fig 1G) [2]. However, as it was further absorbed, the pigment in the trabecular meshwork disappeared but the Sampaolesi line remained and could be used to confirm the history of pigmentary dispersion.

A posterior curvature (concave) of the iris can be detected by slit-lamp examination which is simple and quick. However, a posterior curvature is not a common finding in young children [9], and it can be regarded as an ancillary finding. A pediatric study using anterior segment OCT reported that 24% of the eyes had a posterior curvature of the iris when the subject was fixating a distant object and 65% when the subject was fixating a near object [9]. Thus, standards for the examination protocols must be set to determine the presence of signs that can be used to diagnose PG and PDS.

The mid peripheral iris depigmentation may be difficult to detect in lightly pigmented Caucasians, but it is easy to see in highly pigmented Asians. This is the strongest evidence of pigment dispersion in Asians. However, even if the pigmentation is dense, the depigmentation may not be clear in eyes with only a slight dispersion as it was in Case 2 [10]. We conclude that some kind of image analysis technique needs to be developed to detect the depigmentation accurately.

One possible mechanism for the pigment dispersion is the contact and rubbing of the posterior lobe of the iris against Zinn’s zonule [2]. However, the iris is composed of the anterior lobe, stroma, and the posterior lobe [11] and contact with Zinn’s zonule alone cannot explain the development of the mid peripheral iris transillumination defect and depigmentation of the anterior surface of the iris. In addition, it has been reported that even in PG eyes, the iris is not necessarily in contact with Zinn’s zonule [12].

PG is a disorder that has the opposite characteristics of angle closure (Table 3). In young myopic eyes, all of the tissues of the eye are soft and elastic, the cornea is thin, and the anterior chamber is deep. So, when the eye is squeezed by a blink and the eye moves (Bell’s phenomenon), the pressure in the anterior chamber forces a rapid flow of aqueous humor from the posterior to the anterior chamber. In such eyes, the anterior chamber pressure increases more than the posterior chamber pressure which causes the iris to be posteriorly curved and make contact with Zinn’s zonula fibers [2]. Recent findings showed that the iris curvature can easily change with accommodation and blinking [13], and it has also been reported that the iris can be curved anteriorly or posteriorly even in the same eye [14]. In addition, it has been found that vibratory movements of the eye, such as jogging and cycling, can enhance the pigment dispersion [15, 16]. And one population study showed daily vigorous exercise was associated with higher prevalence of open angle glaucoma [17]. From these findings, it can be inferred that the movement of the iris back and forth causes the pigment dispersion from both the anterior and posterior surface of the iris. Although this needs to be confirmed by experimentation, the clinical findings indicate that the anterior surface of the iris is also the source of the scattered pigments.

**Table 3:** Typical ocular biometric features of old hyperopic primary angle closure eyes (left) and young myopic pigment dispersion eyes (right).

Angle closure		Pigment dispersion
Thick	Cornea	Thin
Hard		Soft
Shallow	Anterior chamber	Deep
Thick	Iris	Thin
Convex		Concave
Thick	Lens	Thin
Hard		Soft
Short	Axial length	Long

PG and PDS develop in school-aged children, and with increasing age, the pigment in the trabecular meshwork is gradually absorbed. The regression phase has occurred when the IOP normalizes. Therefore, in epidemiological studies of adults over 40-years-of-age, eyes that had PG are often diagnosed as NTG [2]. And NTG is a disorder that has the similar ocular biometric features of PG. In addition, because the diagnostic criteria for the classic triad of signs, especially the mid peripheral iris transillumination defect, are not seen in such eyes, PG is not diagnosed using the conventional classic triad of signs especially in Asians. These findings indicate that epidemiological studies using new diagnostic criteria of PG and PDS in Asian individuals under 40-years-of-age are needed.

In epidemiological studies of Asians, myopia was found more frequently in younger age groups and NTG was more frequent in myopic eyes [5, 6]. In addition, NTG with little or no progression was common not only in Asians but also in Caucasians [18, 19]. These facts suggest that these studies most likely included PG patients in the regression phase in the NTG group. Glaucomatous optic atrophy of eyes of school age children with PG may progress when the IOP is elevated, but some PG patients without optic nerve vulnerability hardly progresses when the IOP becomes normalized in the regression phase. In the future, the speed of progression of NTG with a history of pigment dispersion such as mid peripheral iris depigmentation and Sampaolesi line, needs to be investigated.

Ha et al assessed the association between myopia and open-angle glaucoma risk by dose-response meta-analysis and reported that the risk of glaucoma increased more steeply with decreasing spherical equivalent from approximately −6 diopters, and increased more steeply from −8 diopters [20]. The majority of epidemiological studies used in the meta-analysis were not analyzed by OCT, and glaucomatous optic atrophy was determined based on fundus photographs and visual field testing. So, the authors suggest that one reason for the increased risk of developing glaucoma at -8D or higher may be that changes in high myopia or pathological myopia are judged to be glaucomatous optic nerve atrophy. Myopia is a risk factor for pigmented glaucoma, and all subjects in this study were also myopic. One reason for the increased risk of developing glaucoma at -6D or higher in the meta-analysis may be pigmentary glaucoma was determined to be open-angle glaucoma.

From our findings, we propose a triad of signs for the diagnosis of PG and PDS in Asians; the presence of Sampaolesi line, iris concavity, and mid peripheral iris depigmentation. NTG is diagnosed by exclusion, and there is a wide variation in the speed of progression. Therefore, most glaucoma specialists feel the need for a revised classification of NTG. However, some glaucoma specialists may be reluctant to diagnose PG without determining the condition of the eye in childhood. One solution to this problem has been reported to classify open angle glaucoma as primary OAG, exfoliative OAG, and pigmentary OAG [21]. Because a history of pigment dispersion is confirmed by the Sampaolesi line and midperipheral iris depigmentation, we propose that eyes with this history be classified as pigmentary OAG.

This study has several limitations. The first is that it was a hospital-based retrospective study which means that glaucoma specialists diagnosed the glaucoma but did not standardize the examination criteria, and the examined cases may be biased. Secondly, the number of cases was small, and it is necessary to confirm the results in a larger number of cases. The third limitation is that the study was conducted in one region of Japan, and because Asians vary from region to region, epidemiological studies are needed in other regions.

In conclusion, the presence of the Sampaolesi line, iris concavity, and midperipheral iris depigmentation may be appropriate diagnostic findings of PG and PDS, since the specific triad of signs is still under investigation and has not been established to determine the diagnosis. The triad may be useful because if we can classify PG among NTG, including open-angle glaucoma, we may be able to resolve the differences in the types of glaucoma among races.

## Supporting information

S1 Dataset (XLSX)
